# RETRACTED: Shi et al. Surface Hydrophilic Modification for Chip of Centrifugal Microfluidic Immunoassay System. *Micromachines* 2022, *13*, 831

**DOI:** 10.3390/mi16050599

**Published:** 2025-05-21

**Authors:** Yuxing Shi, Peng Ye, Chuang Wang, Kuojun Yang, Jinhong Guo

**Affiliations:** 1 School of Automation Engineering, University of Electronic Science and Technology of China, Chengdu 611731, China; yuxing_shi@163.com (Y.S.); wchu2021@163.com (C.W.); yangkuojun@uestc.edu.cn (K.Y.); 2 The M.O.E. Key Laboratory of Laboratory Medical Diagnostics, The College of Laboratory Medicine, Chongqing Medical University, No. 1 Yixueyuan Road, Chongqing 400016, China

The journal retracts the article titled “Surface Hydrophilic Modification for Chip of Centrifugal Microfluidic Immunoassay System” [1], cited above.

Following publication, the authors contacted the Editorial Office to raise concerns relating to logical errors in the experimental design described in this publication [1].

Adhering to our standard procedure, an investigation was conducted by the Editorial Office and Editorial Board, which confirmed that the article contained a number of significant errors in the methodology. Given the scale and impact that these errors have on the validity of the overall findings, the Editorial Board and authors have determined that the overall findings could not be relied upon and decided to retract this article [1] as per MDPI’s retraction policy (https://www.mdpi.com/ethics#_bookmark30).

This retraction was approved by the Editor-in-Chief of *Micromachines*. The authors agreed to this retraction.
